# Cerebral gray matter volume identifies healthy older drivers with a critical decline in driving safety performance using actual vehicles on a closed-circuit course

**DOI:** 10.3389/fnagi.2025.1462951

**Published:** 2025-05-27

**Authors:** Handityo Aulia Putra, Kaechang Park, Fumio Yamashita

**Affiliations:** ^1^Department of Electrical, Electronics, and Information Engineering, Nagaoka University of Technology, Nagaoka, Japan; ^2^Research Organization for Regional Alliance, Kochi University of Technology, Kochi, Japan; ^3^Department of Decoded Neurofeedback, Computational Neuroscience Laboratory Group, Advanced Telecommunications Research (ATR) International, Kyoto, Japan; ^4^Division of Ultrahigh Field MRI, Institute for Biomedical Sciences, Iwate Medical University, Iwate, Japan

**Keywords:** healthy older drivers, driving safety performance, MRI, regional gray matter volume, machine learning

## Abstract

**Introduction:**

Identifying older drivers at risk of critical decline in driving safety performance (DSP) is essential for traffic safety. Regional cerebral gray matter (GM) volume may serve as a biomarker for such decline, but its predictive value in real-world driving contexts remains unclear.

**Methods:**

We enrolled 94 cognitively healthy older drivers (45 males, 49 females; mean age 77.66 ± 3.67 years) who completed a standardized driving assessment using actual vehicles on a closed-circuit course. DSP was evaluated across six categories: visual search behavior, speeding, indicator signaling, vehicle stability, positioning, and steering. Scores were assigned by a certified driving instructor, with lower scores (<15th percentile) indicating critical DSP decline. Regional GM volumes were quantified using voxel-based morphometry of MRI scans. Feature selection and classification were performed using the Random Forest machine learning algorithm, optimized to identify the most predictive GM regions.

**Results:**

Out of 114 GM regions, eleven were selected as optimal predictors: left angular gyrus, frontal operculum, occipital fusiform gyrus, parietal operculum, postcentral gyrus, planum polare, superior temporal gyrus, and right hippocampus, orbital part of the inferior frontal gyrus, posterior cingulate gyrus, and posterior orbital gyrus. These regions are implicated in attention, spatial cognition, visual processing, and somatosensory integration-functions critical for safe driving. The Random Forest model demonstrated high accuracy and specificity, but moderate precision and recall, limiting immediate real-world application.

**Discussion:**

While regional GM volume shows promise for identifying older drivers at risk of critical DSP decline, predictive performance remains suboptimal for practical implementation. Additional factors, such as neuronal connectivity assessed by functional MRI, may improve predictive accuracy. Nonetheless, MRI-based assessment of brain structure can enhance our understanding of the neural mechanisms underlying driving safety and inform strategies to prevent traffic accidents among older adults.

## Introduction

In countries where the population is aging, the number of traffic fatalities caused by older drivers is increasing year by year, and preventive measures against traffic crashes have become a major social issue. This is especially true in Japan because the proportion of the population aged 65 or older has reached about 30%, making it the fastest-growing country in the world, and this trend is expected to continue until 2060 (Cabinet Office Japan, 2022). Therefore, it is no exaggeration to say that Japan’s measures for older drivers are attracting attention from the world. Japan remains in a position to show fundamental measures that can serve as a valuable example for the world rather than temporary and superficial measures.

As the number of older drivers grows, so does the prevalence of drivers with dementia and mild cognitive impairment (MCI), which may elevate the risk of traffic accidents (Friedland et al., 1988; Brown and Ott, 2004; Mayhew et al., 2006). Since 2017, Japan has mandated cognitive function tests for drivers aged 70 and older when renewing their licenses. However, an official report from the Japanese government indicates that over half of the older drivers involved in accidents had normal cognitive function (Japanese National Police Agency, 2019). This suggests that excluding drivers with dementia or MCI alone will not fully address the problem, as older drivers without these conditions also contribute to traffic accidents (Salthouse, 2000; Abou-Raya and ElMeguid, 2009; Hong et al., 2015; Nishida, 2015). Therefore, it is essential to develop measures that address the decline in driving safety performance (DSP) among older drivers without dementia or MCI (Pavlidis et al., 2016; Talwar et al., 2019; Renge et al., 2020).

DSP is proposed to consist of six categories: visual search behavior, speeding, indicator signals, vehicle stability, positioning, and steering (Park et al., 2022). Our research posits that DSP is regulated by the brain, necessitating an investigation into the brain itself to develop fundamental measures against traffic crashes (Park et al., 2022; Seidler et al., 2010; Sakai et al., 2012; Yamamoto et al., 2020). Magnetic resonance imaging (MRI) allows for the measurement of brain volume data, though its use is limited by the high cost and time required for medical equipment.

Previous research has explored the relationship between brain structure and DSP. For instance, a study by the Toyota Research Center found a significant association between questionnaire scores on DSP and a decrease in the volume of the supplementary motor area in 39 healthy older drivers (Sakai et al., 2012). However, this study did not evaluate DSP in actual vehicles or report on other brain regions. Another study by Keio University investigated only one DSP category—stopping distance due to braking at intersections—in 32 elderly individuals without dementia (Yamamoto et al., 2020). Using machine learning, they identified significant correlations between this limited DSP and the volume of four gray matter (GM) regions. However, this study did not examine other DSP categories or various driving scenarios, such as lane changes or navigating large curves with poor visibility.

In this study, we enrolled 94 older drivers without dementia and examined their DSP using actual vehicles on a closed-circuit course. We evaluated six DSP categories across various driving locations and employed machine learning to identify older drivers with risky driving performance, as defined by their DSP scores. Additionally, we investigated the GM regions involved in this identification process. By addressing these gaps in the literature, our study aims to provide a more comprehensive understanding of the neural basis of DSP in older drivers, potentially contributing to the fundamental development of effectively preventive measures against traffic accidents.

While this approach is particularly relevant in Japan due to the widespread availability of MRI scanners, we recognize that the accessibility of MRI technology may vary in other countries. However, the insights gained from this study could inform future research and policy decisions globally as MRI technology becomes more accessible.

## Materials and methods

### Participants

A total of 94 participants (45 men and 49 women; mean age, 77.66 ± 3. 67 years) without dementia participated in this study. Participants were recruited from the Chuge area of Kochi prefecture in Japan, through local newspapers and television. The gender distribution (45 males, 49 females) closely reflects the general population of older adults in the study area. We did not find significant gender-based differences in our analyses, but future studies with larger sample sizes could further explore potential gender effects. Each participant received an MRI examination and mini-mental state examinations (MMSE) at Tano Hospital, a medical center in the Chugei area. The average MMSE scores were 28.32 ± 1.62 (range, 24–30; median, 29). A dementia specialist (K.P.) interviewed all participants and their families, examined the participants, and ruled out dementia based on MRI findings, MMSE scores, and neuropsychological tests, including the Conversational Assessment of Neurocognitive Dysfunction, a tool newly developed for dementia diagnosis based on daily conversations (Oba et al., 2018). All participants were right-handed and had no cerebrovascular diseases or brain tumors. Massive white matter hyperintensities (WMHs) were also excluded from the enrollment, as WMHs have been reported to deteriorate DSPs (Oba et al., 2022; Park et al., 2013). Participants also received an evaluation of DSP on actual vehicles running on roads at the Aki Driving School located in the Chugei area of Kochi. Inclusion criteria for driving experience and exposure required participants to drive more than twice per week and cover at least 5 km per week to various destinations such as work sites, shops, and hospitals. Professional drivers were excluded from this study.

### Measurement of regional brain volumes

T1-weighted MR images were obtained using the 1.5-Tesla ECHELON Vega system (Hitachi, Tokyo, Japan) with the three-dimensional gradient echo with an inversion recovery sequence. The following scanning parameters were used: repetition time, 9.2 ms; echo time, 4.0 ms; inversion time, 1,000 ms; flip angle, 8°; field of view, 240 mm; matrix size, 0.9375 × 0.9375 mm; slice thickness, 1.2 mm; and the number of excitations, 1. Each image was visually assessed for brain diseases and anomalies, head motion, and artifacts affecting the volumetric measurement. The images were processed and analyzed using the VBM8 toolbox^1^ and other modules implemented in the Statistical Parametric Mapping (SPM) 8 to estimate regional brain volumes.^2^

In brief, the images were segmented into GM, WM, and cerebrospinal fluid space using the maximum a posteriori (MAP) approach (Whitwell, 2009). The segmented GM and WM images were then used to estimate the morphological correspondence between the template image and the participant’s brain using the high-dimensional nonlinear warping algorithm (Ashburner, 2007). The estimated nonlinear warp was inversely applied to an atlas defined in the template space to parcellate the target brain anatomically. The neuronal morphometric atlas was used for the parcellation according to SPM12, with a modification for WM lesions which appeared as incorrect GM segments around the lateral ventricles. The volumes of 114 anatomical regions were calculated as the sum of the correspondent tissue densities in the voxels belonging to each region.

### Evaluation by DSPs

**FIGURE 1:**
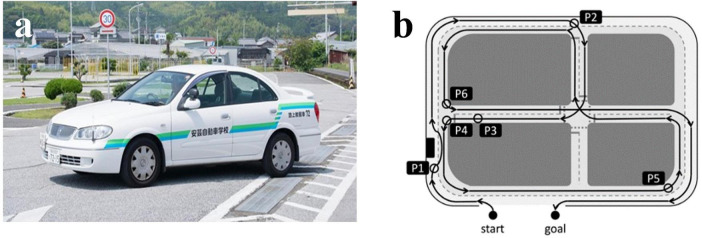
An actual vehicle and a closed-circuit course. **(A)** A view from inside the vehicle. **(B)** Map of the driving course with six rating points. P1, changing line when driving straight; P2, intersection with one right turns; P3, straight course; P4, intersection with one left turn; P5, large curve with poor visibility; P6, another right turn having a stop sign. The corresponding author owns the copyright of the photography.

Actual vehicle-driving experiments were performed on a closed-circuit course, officially designated for renewing driving licenses for older drivers by the National Police Agency (The Driver’s License Skill Test Implementation Standard), in the Aki Driving School in the Chugei area, Kochi, Japan (Figure 1A). In the present test, six locations on the driving course were selected for rating. These locations included changing lines when driving straight (Figure 1B, P1), changing line when driving straight; P2, intersection with one right turn; P3, straight course; P4, intersection with one left turn; P5, large curve with poor visibility; P6, another right turn having a stop sign.

The six locations were selected to represent a variety of driving scenarios commonly encountered by older drivers. These include straight driving, turns, intersections, and areas with poor visibility. All participants followed a predetermined sequence of driving routes on the closed-circuit course without breaks between rounds. This approach ensured consistency across evaluations and comparability with prior studies using the same protocol (Park et al., 2024). While this fixed order minimizes variability in assessment conditions, it may introduce potential order effects or fatigue-related biases. Future studies could explore randomized route sequences or incorporate breaks between rounds to reduce these biases while maintaining standardized evaluation procedures.

A Toyota-made four-wheeled 1,400-cc vehicle (COMFORT) was used. The typical speed of the vehicles on the closed-circuit course ranges from 20 to 50 km/h, and approximately 20 min is taken to complete a circuit. An official driving instructor can accomplish the evaluation after showing participants how to drive, as a good sample of DSP. No further driving events were included in the test. In the advanced stage of the test, a qualified driving instructor drove around the course, demonstrating good driving performance, with a participant sitting in the seat next to the instructor. Then, the participant drove with the evaluating instructor sitting in the passenger’s seat. The official instructor rated the driving skills of each participant using the previously described method (Supplementary Table S1) (Park et al., 2024). They responded to the items using a three-point scale: (1) poorly done; (2) normally done; and (3) well done. These rating scores at six locations were then calculated as the “overall evaluation” by assessing the six categories: DSP1, “visual search behavior (safety recognition with head movement);” DSP2, “speeding (choice of vehicle speed);” DSP3, “signaling (timely and appropriate usage of the indicator);” DSP4, “vehicle stability (acceleration and braking without knocking and completely pulling up in front of the stop line);” DSP5, “positioning (vehicle movement along the radius of the curvature at intersections without large or small turns);” DSP6, “steering (smooth handling with appropriate starting and ending).” The six categories of driving safety performance (DSP1-DSP6) were based on previous research in traffic safety and recommendations from experienced driving instructors. These categories encompass key aspects of safe driving behavior that are particularly relevant for older adults, as previously described (Park et al., 2024).

Larger scores indicated stronger compliance with the Road Traffic Act. The average value of the summed scores at the six locations for the two rounds of the course was calculated for the DSPs. To minimize potential bias, the driving instructor underwent standardized training in assessment procedures. However, we acknowledge that some subjectivity may remain. Future studies could benefit from multiple raters and the calculation of inter-rater reliability.

### Statistical analysis

To account for potential gender differences in brain structure, independent samples *t*-tests were conducted to compare normalized brain volumes between male and female participants. Significant differences in frontal and parietal volumes were identified (see “Results”), and gender was subsequently included as a covariate in all machine learning models to ensure robustness across gender groups.

### Machine learning analysis

The machine learning analysis was conducted using the scikit-learn library in Python, following a systematic process to ensure rigor and reproducibility. The dataset was initially loaded and preprocessed by removing specific columns deemed irrelevant for the analysis. Feature scaling was performed using MinMaxScaler to normalize the data within the range of 0–1, ensuring that no single feature dominated the machine learning models (Pedregosa et al., 2011; de Amorim et al., 2023).

The sample size for this study was 94 participants, which is larger than previous comparable studies in this field (Sakai et al., 2012; Yamamoto et al., 2020). However, machine learning models analyzing neuroimaging data ideally require 100+ participants to achieve stable feature selection (Vabalas et al., 2019).

To mitigate the potential limitations of our sample size, and to enhance the robustness of the model evaluation, bootstrapping was employed. This involved 100 iterations where, in each iteration, a bootstrap sample of the dataset was created and subsequently split into training (70%) and testing (30%) sets (Huang and Huang, 2023). This technique allows for a more reliable estimation of model performance across multiple subsamples of the data.

Additionally, dimensionality reduction in the form of Feature selection was conducted using LASSO (Least Absolute Shrinkage and Selection Operator) regression with 5-fold cross-validation (Tibshirani, 1996) to balance model complexity with the available sample size. Given the 94 samples in our dataset, we constrained the LASSO to select between 7 and 17 features. This range was chosen based on the standard rule of thumb of having approximately 10 samples for each feature, which helps to prevent overfitting while still capturing important predictors (Friedman et al., 2010). Only the top features of the highest importance were retained for further analysis.

To define the critical decline in driving safety performance, we employed a systematic, data-driven process to determine the optimal percentile threshold. The 15th percentile threshold was selected based on the following steps:

1. Iterative threshold testing: We evaluated multiple percentile thresholds (10, 15, 20, and 25%) to identify the optimal split for our dataset.
2. Bidirectional analysis: For each threshold, we created binary groups using both top-X% vs. the rest and bottom-X% vs. the rest of the data.
3. Model development: We developed Random Forest models for each grouping, using 5-fold cross-validation to ensure robustness.
4 . Performance comparison: We compared model performance across thresholds using multiple metrics:
• Bottom 15%: Accuracy = 0.89, Precision = 0.72, Recall = 0.64, F1-score = 0.62, AUC = 0.85.
• Other thresholds: Accuracy = 0.82–0.86, Precision = 0.65–0.70, Recall = 0.58–0.62, F1-score = 0.55–0.60, AUC = 0.78–0.82.
5. Consistency check: We found that the bottom 15% threshold consistently outperformed other splits across all six Driving Safety Behavior (DSB) categories.
6. Validation: We used bootstrapping (100 iterations) to validate the stability of our results, finding consistent performance (AUC variation: ± 2%) for the 15% threshold.

While this threshold is not a standard statistical cutoff, it provided the most meaningful and stable separation in our dataset for identifying drivers with potentially critical declines in performance. This data-driven approach, combined with the expertise of driving instructors, offers a balance between statistical rigor and practical relevance in the context of driving safety assessment.

To address the class imbalance that is present in the dataset (14 vs. 84 participants), we employed the Synthetic Minority Over-sampling Technique (SMOTE). This technique oversamples the minority class in the training data to achieve balanced classes, which helps to balance the classes and improve the model’s ability to learn from the underrepresented group (Q. Chen et al., 2022).

To identify the optimal classification algorithm for predicting critical decline in DSP, we conducted a comprehensive comparison of nine machine learning algorithms: Logistic Regression, Decision Tree, Random Forest, Gradient Boosting, k-Nearest Neighbors, Naive Bayes, Support Vector Machine, Neural Network, and AdaBoost. All models were evaluated using 10-fold bootstrapping (*n* = 280 per classifier), with performance assessed across multiple metrics including accuracy, precision, recall, F1-score, and ROC-AUC. To address the class imbalance in our dataset (14 vs. 84 participants), we applied the Synthetic Minority Over-sampling Technique (SMOTE) during model training. Statistical comparisons between model performances were conducted using ANOVA with *post-hoc* tests, using Support Vector Machine as the reference classifier. The Random Forest classifier was ultimately selected based on its superior performance across these metrics.

While we acknowledge the limitations of our small sample size, the use of bootstrapping and cross-validation helps to maximize the use of our available data and provides a more robust estimate of model performance. However, we recognize that these results should be interpreted with caution, and future studies with larger sample sizes are needed to confirm our findings.

### Ethics statement

This study was conducted under the “Ethics Guideline for Medical and Health Research Involving Human Subjects” based on the Declaration of Helsinki. All participants signed a formal agreement outlining that the experimental data would only be used for scientific study and that the results would ensure anonymity. Written informed consent was obtained from all participants. This study was approved by the institutional review board at Kochi University of Technology (Application no. C4-3).

## Results

### Determination of critical decline in driving safety performance

**FIGURE 2:**
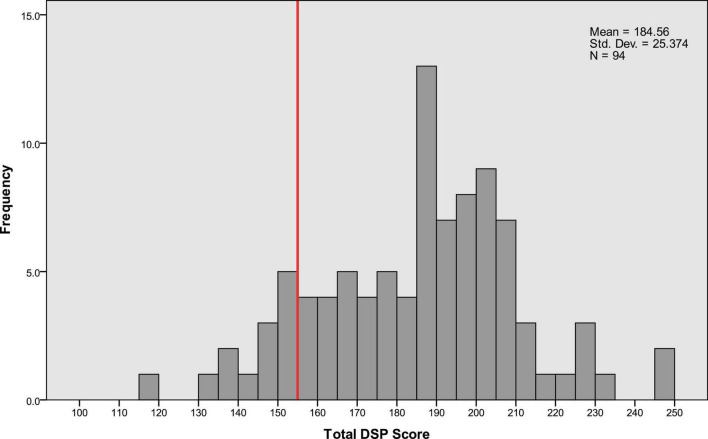
Distribution of the total driving safety performance (DSP) scores of the participants. The total DSP score is the sum of scores from DSP1 to DSP6. This score was used to build the Random Forest model in this study. The red line on the x-axis indicates the 15th percentile threshold, marking the boundary for the lowest 15% of scores.

We analyzed the distribution of DSP scores across six categories: visual search behavior, speeding, signaling, vehicle stability, positioning, and steering (Supplementary Figure S1). A threshold for critical decline in DSP was established at the 15th percentile of total DSP scores (Figure 2). This threshold was corroborated by official driving instructors based on their extensive experience in license renewal for older drivers. The use of this threshold aligns with previous research suggesting that older drivers may have increased risk in complex driving situations due to age-associated changes in attention and cognitive decline (Henderson et al., 2013).

### Participant characteristics

Participants were divided into two groups based on the 15th percentile threshold of total DSP scores: the lower DSP group (scores below the 15th percentile) and the higher DSP group (scores above the 15th percentile). The demographic and brain volume metrics for both groups are summarized in Table 1. There were no significant differences between the groups in terms of age (*p* = 0.915), MMSE scores (*p* = 0.174), gray matter volume ratio (*p* = 0.713), white matter volume ratio (*p* = 0.654), or total brain volume to intracranial volume ratio (*p* = 0.702).

**TABLE 1:** Demographic and brain volume metrics for different DSP score percentiles.

	Lower 15% percentile of DSP score	Upper 85% percentile of DSP score	All participants
*N*	14	80	94
Age	77.86 ± 4.61	77.63 ± 3.52	77.66 ± 3.67
MMSE score	28.36 ± 2.13	28.31 ± 1.52	28.32 ± 1.62
Total DSP score	143.57 ± 10.67	191.74 ± 19.73	184.56 ± 25.37
GM volume/ICV	0.4030 ± 0.0267	0.3909 ± 0.0264	0.3928 ± 0.0266
WM volume/ICV	0.3683 ± 0.0229	0.3831 ± 0.0230	0.3808 ± 0.0235
Brain volume/ICV	0.7713 ± 0.0209	0.7740 ± 0.0235	0.7736 ± 0.0230

This table compares demographic data and brain volume metrics between participants in the lower 15th percentile of DSP scores, the upper 85th percentile of DSP scores, and all participants. MMSE, Mini-Mental State Examination; DSP, Driving Safety Performance; GM, Gray Matter; WM, White Matter; ICV, Intracranial Volume.

Gender-based analysis of normalized brain volumes revealed (Table 2) a significant difference between male and female participants in total frontal volume (*p* = 0.012) and a marginally significant difference in total parietal volume (*p* = 0.066). No significant gender differences were observed in other global or regional brain measurements. To account for these differences, gender was included as a covariate in subsequent analyses examining the relationship between brain structure and driving performance.

**TABLE 2:** Gender-based comparison of normalized brain volumes and age among participants.

Parameters	Mean (male)*	Mean (female)*	*t*-statistic	*p*-value**	Cohen’s *d*
Age	77.67	77.65	0.018	0.986	0.004
Total gray matter volume	0.3885	0.3967	−1.492	0.139	−0.308
Total white matter volume	0.3835	0.3784	1.027	0.307	0.212
Total cerebrospinal fluid volume	0.228	0.2249	0.665	0.507	0.137
Total brain volume	0.7719	0.7751	−0.665	0.507	−0.137
Total frontal volume	0.0953	0.0996	−2.552	0.012	−0.527
Total temporal volume	0.0703	0.0702	0.048	0.962	0.01
Total parietal volume	0.0591	0.0613	−1.863	0.066	−0.385
Total occipital volume	0.0444	0.0449	−0.559	0.577	−0.116

This table presents the mean values, *t*-statistics, *p*-values, and Cohen’s *d* effect sizes for comparisons between male (*n* = 45) and female (*n* = 49) participants across various brain volume metrics normalized by intracranial volume (ICV).

*All brain volumes are normalized by intracranial volume.

**p-values are two-sided *p*-value.

### Comparison of machine learning models for DSP prediction

A systematic comparison of nine machine learning algorithms revealed significant differences in F1-scores [*F*(8, 2,511) = 83.156, *p* < 0.001, partial η^2^ = 0.209]. As shown in Tables 3, 4, the Random Forest classifier achieved superior overall performance with the highest F1-score [0.558 ± 0.084, 95% CI (0.025, 0.054)] and ROC-AUC (0.805 ± 0.051) compared to other algorithms. This was followed by Gradient Boosting (F1 = 0.549 ± 0.080) and AdaBoost (F1 = 0.546 ± 0.083). The Random Forest model demonstrated particularly strong precision (0.641 ± 0.123) and specificity (0.94), though with moderate recall/sensitivity (0.573 ± 0.091).

**TABLE 3:** Performance comparison of multiple different classifiers with SMOTE balancing.

**Classifier comparison with SMOTE balancing**				
Classifier	Precision	Recall	F1-score (95% CI)	ROC-AUC
AdaBoost	0.5791 ± 0.1081 **	0.599 ± 0.0895	0.5458 ± 0.0825 [0.013, 0.042] **	0.7631 ± 0.0552 *
Decision Tree	0.547 ± 0.0948	0.6021 ± 0.0826	0.5333 ± 0.0763 [0, 0.029] *	0.7409 ± 0.0414 *
Gradient Boosting	0.5714 ± 0.1019 *	**0.6083 ± 0.0815** *	0.5489 ± 0.0796 [0.016, 0.045] **	0.7756 ± 0.0524 **
k-Nearest Neighbors	0.4989 ± 0.1052 **	0.6079 ± 0.1053	0.5174 ± 0.0942 [−0.016, 0.013]	0.7764 ± 0.0504 **
Logistic Regression	0.4417 ± 0.1184 **	0.5826 ± 0.0882	0.466 ± 0.0951 [−0.067, −0.038] **	0.7185 ± 0.0676 **
Naive Bayes	0.4049 ± 0.1128 **	0.5095 ± 0.0928 **	0.408 ± 0.0819 [−0.125, −0.096] **	0.6986 ± 0.0621 **
Neural Network	0.5261 ± 0.1178	0.6021 ± 0.0903	0.5222 ± 0.0964 [−0.011, 0.018]	0.7449 ± 0.0649
Random Forest	**0.6413 ± 0.1226** **	0.5732 ± 0.091 *	**0.5579 ± 0.0836 [0.025, 0.054]** **	**0.8045 ± 0.0507** **
Support Vector Machine	0.5437 ± 0.1349 [+]	0.589 ± 0.0869 [+]	0.5186 ± 0.0945 [reference] [+]	0.753 ± 0.0694 [+]

This table presents the precision, recall, F1-score, and ROC-AUC score of various machine learning models with SMOTE balancing. All models trained using SMOTE to address class imbalance (14 vs. 84 participants). Results based on 10-fold bootstrapping (*n* = 280 per classifier). Overall ANOVA: F(8, 2511) = 83.156, *p* < 0.001, partial η^2^ = 0.209. Bold values indicate the highest value within each column (performance metric) in the table.

*p < 0.05,

**p < 0.01 compared to Support Vector Machine (reference classifier). [+] reference classifier. The 95% confidence intervals (CI) for F1-scores represent the difference between each classifier and the reference classifier (Support Vector Machine).

**TABLE 4:** Performance comparison of multiple different classifiers without SMOTE balancing.

**Classifier comparison without SMOTE balancing**				
Classifier	Precision	Recall	F1-score (95% CI)	ROC-AUC
AdaBoost	0.589 ± 0.1231 **	0.4521 ± 0.1162 **	0.4743 ± 0.1084 [0.0525, 0.0909] **	0.7536 ± 0.0544 **
Decision Tree	0.5291 ± 0.1086	**0.4828 ± 0.1018** **	0.4693 ± 0.0948 [0.0474, 0.0859] **	0.7321 ± 0.0422
Gradient Boosting	0.6002 ± 0.1098 **	**0.4828 ± 0.1025** **	0.4981 ± 0.0938 [0.0762, 0.1147] **	0.7629 ± 0.0506 **
k-Nearest Neighbors	0.6238 ± 0.1185 **	0.4775 ± 0.1158 **	0.5045 ± 0.1066 [0.0826, 0.1211] **	0.7805 ± 0.0538 **
Logistic Regression	0.4733 ± 0.1627 **	0.3552 ± 0.1478 *	0.3722 ± 0.1424 [−0.0497, −0.0112] *	0.7197 ± 0.0685
Naive Bayes	0.4505 ± 0.1415 **	0.3345 ± 0.1194 **	0.3459 ± 0.1152 [−0.076, −0.0375] **	0.7102 ± 0.0639 **
Neural Network	0.5169 ± 0.1391	0.4389 ± 0.1409 **	0.4402 ± 0.1324 [0.0183, 0.0568] **	0.7306 ± 0.0696
Random Forest	**0.6994 ± 0.1298** **	0.4595 ± 0.1016 **	**0.5195 ± 0.0987 [0.0977, 0.1361]** **	**0.7958 ± 0.0511** **
Support Vector Machine	0.5166 ± 0.162 [+]	0.3816 ± 0.1424 [+]	0.4027 ± 0.1396 [reference] [+]	0.7281 ± 0.0803 [+]

This table present the precision, recall, F1-score, and ROC-AUC score of various machine learning models without the SMOTE balancing. All models trained using SMOTE to address class imbalance (14 vs. 84 participants). Results based on 10-fold bootstrapping (*n* = 280 per classifier). Overall ANOVA: *F*(8, 2,511) = 83.156, *p* < 0.001, partial η^2^ = 0.209. Bold values indicate the highest value within each column (performance metric) in the table.

*p < 0.05,

**p < 0.01 compared to Support Vector Machine (reference classifier). [+] reference classifier. The 95% confidence intervals (CI) for F1-scores represent the difference between each classifier and the reference classifier (Support Vector Machine).

Despite implementing SMOTE to address class imbalance, precision and recall values remained moderate across all models. However, adding SMOTE increases the performance of the model compared to without using SMOTE. This limitation can be attributed to several factors: (1) sample size constraints relative to the number of predictors, (2) persistent challenges in classifying the minority class despite oversampling, (3) complex non-linear relationships between brain volumetric data and driving performance, and (4) inherent variability in real-world driving assessments.

To optimize model robustness given these constraints, we implemented multiple improvement strategies including bootstrapping (100 iterations), dimensionality reduction via LASSO regression with cross-validation, and algorithm-specific hyperparameter tuning. These measures collectively improved model stability while maintaining the balance between precision and recall.

### Prediction performances using Random Forest

The Random Forest model was evaluated with different numbers of features selected by the LASSO method. Table 5 presents the values of accuracy, precision, recall/sensitivity, and F1 scores for count of features ranging from 7 to 17. The best predictive performance was achieved using 12 features, including sex, with the following mean metrics: accuracy (0.89), precision (0.72), recall/sensitivity (0.64), F1 score (0.62), ROC-AUC (0.85), specificity (0.94), and cross-validation score (0.95). These results indicate a strong overall performance of the model in identifying drivers with critically declined DSP, with particularly high accuracy and specificity. However, the relatively lower recall/sensitivity suggests that the model may have some difficulty in identifying all cases of critically declined DSP.

**TABLE 5:** Performance metrics of Random Forest model with different feature counts.

Feature count	Accuracy		Precision		Recall		F1 score		ROC-AUC		Specificity		Cross-validation	
	Mean	Std	Mean	Std	Mean	Std	Mean	Std	Mean	Std	Mean	Std	Mean	Std
7	0.87	0.08	0.74	0.23	0.56	0.26	0.59	0.19	0.85	0.09	0.90	0.11	0.95	0.07
8	0.89	0.08	0.70	0.34	0.64	0.27	0.60	0.21	0.87	0.09	0.94	0.10	0.95	0.09
9	0.89	0.09	0.69	0.29	0.57	0.26	0.59	0.22	0.79	0.19	0.94	0.07	0.96	0.03
10	0.81	0.13	0.58	0.29	0.56	0.24	0.50	0.19	0.75	0.17	0.86	0.18	0.91	0.12
11	0.88	0.10	0.73	0.34	0.60	0.32	0.61	0.30	0.80	0.18	0.94	0.12	0.95	0.07
12	**0.89**	0.10	**0.72**	0.31	**0.64**	0.30	**0.62**	0.24	**0.85**	0.13	**0.94**	0.12	**0.95**	0.08
13	0.84	0.12	0.62	0.38	0.51	0.33	0.47	0.28	0.78	0.14	0.91	0.15	0.94	0.09
14	0.87	0.12	0.61	0.35	0.61	0.30	0.56	0.29	0.84	0.21	0.91	0.14	0.95	0.06
15	0.86	0.11	0.64	0.33	0.66	0.28	0.57	0.21	0.83	0.15	0.88	0.13	0.94	0.10
16	0.83	0.15	0.49	0.32	0.52	0.33	0.45	0.26	0.80	0.15	0.87	0.18	0.93	0.10
17	0.90	0.06	0.67	0.37	0.58	0.32	0.54	0.26	0.84	0.17	0.95	0.07	0.96	0.04

This table presents the performance metrics of the Random Forest model for various feature counts, including accuracy, precision, recall, F1 score, ROC-AUC, specificity, and cross-validation. DSP, Driving Safety Performance; ROC-AUC, Receiver Operating Characteristic Area Under Curve. Bold values indicate the highest value within each column (performance metric) in the table.

### Statistical analysis of brain-behavior relationships

**FIGURE 3:**
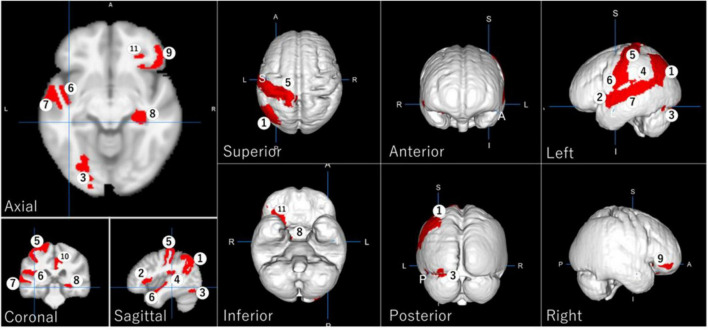
Regional gray matter areas involved in driving safety performance (DSP) as selected by LASSO, providing the best Random Forest model with the highest evaluation results. The identified regions are: (1) left angular gyrus, (2) left frontal operculum, (3) left occipital fusiform gyrus, (4) left parietal operculum, (5) left postcentral gyrus, (6) left planum polare, (7) left superior temporal gyrus, (8) right hippocampus, (9) right orbital part of the inferior frontal gyrus, (10) right posterior cingulate gyrus, and (11) right posterior orbital gyrus.

Eleven GM regions were selected by Random Forest with LASSO. According to projecting views, Figure 3 shows the left angular gyrus and the left post central gyrus in the superior view; the left occipital fusiform gyrus, the right hippocampus, and the right posterior orbital gyrus in the inferior view; the left angular gyrus and the left inferior occipital gyrus in the posterior view; the left angular gyrus, the left frontal operculum, the left occipital fusiform gyrus, the left parietal operculum, the left postcentral gyrus, the left planum polare, and the left superior temporal gyrus in the left view; the right orbital part of the inferior frontal gyrus in the right view.

These regions are involved in various cognitive functions crucial for driving, including attention, spatial cognition, visual processing, memory, and decision-making. The identification of these specific regions aligns with previous research highlighting the importance of visual processing and cognitive functions in driving performance (Depestele et al., 2020; Kline et al., 1992).

Bootstrap analysis (Table 6) revealed two brain regions with high selection stability: left postcentral gyrus (70.75%) and right posterior orbital gyrus (54.38%). Inter-group *t*-tests (Table 7) identified three regions with statistically significant volume differences between critical decline and non-decline groups: right posterior orbital gyrus (*p* = 0.0012), right hippocampus (*p* = 0.0162), and left postcentral gyrus (*p* = 0.0414).

**TABLE 6:** LASSO coefficients of the selected brain regions for the model with best performance.

Brain region	LASSO coefficient	Selection frequency (%)
Left angular gyrus	−0.12136	23.25
Left frontal operculum	0.12688	2.625
Left occipital fusiform gyrus	−0.17472	32
Left parietal operculum	0.41	10.125
Left postcentral gyrus	0.42	70.75
Left planum polare	0.18064	8.625
Left superior temporal gyrus	−0.312	12.5
Right hippocampus	0.1584	18
Right orbital part of the inferior frontal gyrus	−0.35048	41.375
Right posterior cingulate gyrus	0.25512	13.628
Right posterior orbital gyrus.	0.30072	54.375

This table presents the LASSO coefficient and selection frequency of the selected features.

**TABLE 7:** Inter-group difference test on the selected features.

Brain region	*t*-test mean difference	*t*-test 95% CI of difference	*t*-statistic	*p*-value	Cohen’s *d*
Left angular gyrus	0.0963	[−0.3298, 0.5223]	0.4487	0.6547	0.1300
Left frontal operculum	−0.0230	[−0.0978, 0.0518]	−0.6104	0.5431	−0.1768
Left occipital fusiform gyrus	0.1103	[−0.0309, 0.2515]	1.5516	0.1242	0.4495
Left parietal operculum	−0.0409	[−0.1509, 0.0691]	−0.7376	0.4626	−0.2137
Left postcentral gyrus	−0.4411	[−0.8645, −0.0176]	−2.0688	0.0414	−0.5993
Left planum polare	−0.0290	[−0.1311, 0.0731]	−0.5636	0.5744	−0.1633
Left superior temporal gyrus	−0.0035	[−0.2765, 0.2695]	−0.0253	0.9799	−0.0073
Right hippocampus	−0.1993	[−0.3608, −0.0377]	−2.4502	0.0162	−0.7098
Right orbital part of the inferior frontal gyrus	−0.0498	[−0.1303, 0.0307]	−1.2294	0.2220	−0.3562
Right posterior cingulate gyrus	−0.1012	[−0.2564, 0.0539]	−1.2961	0.1982	−0.3755
Right posterior orbital gyrus.	−0.1576	[−0.2466, −0.0687]	−3.6543	0.0012	−0.8033

This table presents mean differences, *t*-test score, *p*-value, and Cohen’s *d* value of the features that are selected by the LASSO feature selection.

## Discussion

Brain functions are generally known to be localized according to anatomical structures such as GM regions. Increasing evidence suggests that not only local specialization but also neural connectivity between these regions—organized as large-scale functional networks—regulates higher brain functions and thereby complex human behaviors such as driving a car (Ju, 2023; Thomas Yeo et al., 2011). Because 1.5 Tesla MRI is widely available in Japan and is popularly used in brain health checkups for early detection of unruptured cerebral aneurysms, it is not so difficult to obtain regional GM volumetric data using conventional 1.5 Tesla MRI. On the other hand, neural connectivity can be measured as functional data only when using 3 Tesla MRI which is used in research institutes or medical centers and is not widely available. In this study, we explored the prediction of risky driving performance using regional GM volume with 1.5 Tesla MRI, intending to implement near future this approach in driver’s license renewal for older drivers throughout Japan. Based on our literature review, only two manuscripts except for ours have already described the relationship between regional GM volume data and driving behavior assessment (Sakai et al., 2012; Yamamoto et al., 2020).

Our findings align with established sexual dimorphism in brain structure, particularly in frontal and parietal regions (Ruigrok et al., 2014). The inclusion of gender as a covariate ensures that our model accounts for these anatomical differences, strengthening the generalizability of our results across genders. The preserved relationship between gray matter volume and DSP after controlling for gender suggests that structural brain markers of driving performance are robust to sex-based variation.

Furthermore, this study reports the first time that machine learning methods have been used to assess six categories of DSP in a real vehicle on a closed-circuit course comprehensively enough to assess driver’s license renewal. The results further highlight the utility of brain structural data, such as regional GM volume, in assessing DSP in older drivers.

### Model performance analysis

In this study, we systematically compared nine machine learning algorithms to identify the optimal classifier for predicting critical DSP decline. Random Forest achieved superior performance compared to other models such as Support Vector Machine (Yamamoto et al., 2020), with an F1-score of 0.558 ± 0.084 and ROC-AUC of 0.805 ± 0.051. Random Forest achieved superior performance using twelve features, with accuracy (0.89 ± 0.10), precision (0.72 ± 0.31), recall (0.64 ± 0.30), F1-score (0.62 ± 0.24), ROC-AUC (0.85 ± 0.13), and specificity (0.94 ± 0.12). Bootstrapping with 100 iterations and dimensionality reduction via LASSO regression helped mitigate overfitting risks while improving model stability.

Statistical analysis revealed significant predictors such as the left postcentral gyrus (*p* = 0.0414, Cohen’s *d* = −0.60) and right posterior orbital gyrus (*p* = 0.0012, *d* = −0.80), which were selected with high frequency during bootstrap iterations (70.8 and 54.4%, respectively). Supplementary predictors such as the hippocampus (18%) and occipital fusiform gyrus (32%) showed moderate effect sizes but contributed meaningfully to overall model performance. These findings suggest that driving performance relies on both core neural substrates and distributed networks.

The predictive performance indicates that the precision and recall/sensitivity remain relatively low for practical use such as the assessment of driver’s license renewal for older people while accuracy, ROC-AOC, specificity, and cross-validation reached satisfactory levels. To address this, we plan to examine leukoaraiosis (LA), ischemic lesions in cerebral white matter, which can also be measured by 1.5 Tesla MRI, before exploring functional data from 3 Tesla MRI. LA has been frequently diagnosed among the elderly and has already been significantly associated with traffic crashes and wrong entries on highways (Park et al., 2013; Park and Nakagawa, 2023). Furthermore, a recent study has shown that parietal and occipital LA degrade the DSP of older drivers operating actual vehicles on a closed-circuit course under the same conditions as the present study (Oba et al., 2022). Investigating LA is expected to improve prediction performance because LA is regarded to disrupt neural networks within cerebral white matter (Michely et al., 2018) and may be associated to the degradation of DSP (Oba et al., 2022).

In this study, 11 GM regions were selected using the Random Forest method. The functional roles of these regions are plausible for involvement in DSP as follows: the angular gyrus is involved in attention and spatial cognition (Studer et al., 2014), which are critical for navigating complex driving environments, maintaining awareness of surrounding vehicles, and processing spatial information for lane changes and turns; the frontal operculum play a role in visual emotion detection (Kumar et al., 2009) and visuo-motor performance (Quirmbach and Limanowski, 2022), which may be important for error detection and performance adjustment during driving; the occipital fusiform gyrus is responsible for higher processing of visual information (Uono et al., 2017), which may be important for error detection and performance adjustment during driving; the parietal operculum act as an integration center within a multimodal network (Fornia et al., 2024), which may be important for error detection and performance adjustment during driving; the postcentral gyrus involved in somatosensory processing (DiGuiseppi and Tadi, 2024), necessary for the tactile feedback required during driving, such as feeling the steering wheel and pedals; the planum polare is associated with complex auditory information processing (Griffiths and Warren, 2002), important for hearing and responding to traffic sounds and auditory cues; the superior temporal gyrus involved in sound recognition and speech processing (Yi et al., 2019), enabling drivers to understand spoken instructions and communicate effectively; the hippocampus plays a role in memory and emotion (Immordino-Yang and Singh, 2013), crucial for recalling routes and managing stress while driving; the orbital part of the inferior frontal gyrus is a part of the language processing network (Du et al., 2020), important for reading road signs and understanding verbal instructions; the posterior cingulate gyrus involved in encoding and retrieval of episodic memories (Natu et al., 2019), helping drivers recall specific driving experiences and apply learned behaviors; the posterior orbital gyrus associated with integrating emotions and memories related to sensory experiences (Kim et al., 2017), which may influence decision-making during driving.

Taken together, these regions map onto multiple large-scale brain networks—including the somatosensory-motor, default mode, and frontoparietal control networks—highlighting that safe driving performance in older adults depends on the integrity and coordination of distributed neural systems (Thomas Yeo et al., 2011).

Furthermore, the statistical analyses revealed convergent evidence for the importance of specific brain regions in predicting driving performance. Particularly notable is the left postcentral gyrus, which demonstrated both high selection stability (70.75%) and statistically significant volume differences between performance groups (*p* = 0.0414, *d* = −0.5993). Similarly, the right posterior orbital gyrus showed high selection consistency (54.38%) and the strongest inter-group difference (*p* = 0.0012, *d* = −0.8033), suggesting its critical role in maintaining driving safety performance. The consistent identification of these regions across different statistical approaches strengthens confidence in their relevance to driving performance in older adults.

The involvement of these diverse brain regions underscores the complexity of driving as a cognitive task, requiring the integration of multiple sensory modalities, attention, memory, and decision-making processes. Thus, the findings suggest that driving involves neural connectivity within the brain and may indicate the feasibility of our research approach in implementation with brain structural data through 1.5 Tesla MRI. Furthermore, understanding the roles of these regions in driving performance could have implications for assessing fitness to drive and developing targeted interventions to improve driving skills.

A research team from Keio University previously reported four GM regions after machine learning analysis: the left upper part of the precentral sulcus, the left intermediate sulcus, the right orbital part of the inferior frontal gyrus, and the right superior frontal sulcus (Yamamoto et al., 2020). They used the Support Vector Machine method and focused on braking operations at intersections. The only common region between their results and ours is the right orbital part of the inferior frontal gyrus, suggesting its important role in the DSP of older drivers beyond its known function in the language processing network. However, the other ten GM regions that we reported in this study did not match those reported by them. Our study adopted different conditions, including the Random Forest method, six categories of DSP including braking operations, and evaluations at various locations including intersections on a closed-circuit course. Therefore, caution must be required when identifying the brain regions involved in DSP, as even slight differences in experimental conditions may have a significant effect on DSP.

The results of this study should be interpreted with caution due to several limitations. First, the number of participants may be relatively small. However, to the best of our knowledge, the only comparable study is conducted by a research team at Keio University and Toyota. These studies had even smaller sample sizes, such as the Toyota study with 39 older participants (Sakai et al., 2012) and the Keio University study with 32 participants (Yamamoto et al., 2020). While our sample size of 94 participants is larger than previous studies in this field, it remains a limitation for generalizability, particularly for machine learning models that require larger datasets for stable feature selection (Sarica et al., 2017; Vabalas et al., 2019). While VBM provides robust volumetric estimates, longitudinal stability of measurements in aging populations requires further study. Potential drift in MRI scanner stability over time was mitigated through regular phantom calibration. The relatively low precision and recall metrics observed in our Random Forest model are likely influenced by this limitation. Although bootstrapping and dimensionality reduction helped mitigate overfitting risks, future studies should aim to include larger samples to validate our findings further. Increasing sample sizes would enhance statistical power and allow for more reliable identification of brain regions with smaller effect sizes.

Secondly, this study was conducted on a closed-circuit course under the supervision of an instructor, which may affect DSPs compared to free driving on general roads. A true DSP must be evaluated in privately owned cars on normal roads without instructors. We acknowledge that the closed-circuit environment may not fully reflect real-world driving conditions. However, this controlled setting allows for standardized assessment and minimizes risks to participants. Future research could explore ways to incorporate more realistic driving scenarios while maintaining safety. Third, all participants were over 70 years old, limiting the generalizability of the findings. We recognize that our study is limited by the lack of comparison data from different age groups and regions. Future research should aim to include a broader range of participants to enhance the generalizability of the findings. The relationship between brain structure and driving performance may be universal across all ages. To validate the results, we plan to evaluate DSPs through MRI measurements for middle-aged and young drivers.

While our study focused on gray matter volume, we recognize the importance of other factors such as functional connectivity. Future studies should aim to incorporate multiple neuroimaging modalities to provide a more comprehensive understanding of the neural basis of driving safety in older adults. Moreover, future studies could benefit from using regression models to examine the continuous relationship between gray matter volume and driving safety performance. This approach would allow for a more nuanced understanding of the association and could help identify potential thresholds for intervention. Additionally, exploring alternative statistical methods could provide deeper insights into the complex relationship between brain structure and driving performance, potentially revealing non-linear associations or interaction effects that our current methodology may have overlooked.

## Conclusion

In conclusion, while structural brain metrics show promise for predicting driving safety in older adults, further refinement of methods and expansion to other neuroimaging markers are needed before practical implementation. This study provides a foundation for continued work toward developing brain-based screening tools to promote safe driving and mobility in aging populations.

## Data Availability

The data supporting the findings of this study are available from the corresponding author upon reasonable request. Researchers interested in accessing the data must submit a formal request to the corresponding author, which will be reviewed by the host institution for ethical and privacy considerations. Only de-identified (blinded) data, with all personally identifying information removed, will be made available to qualified researchers.

## Footnotes

1
http://www.neuro.uni-jena.de/vbm8/VBM8-Manual.pdf

2
https://www.fil.ion.ucl.ac.uk/spm/
