# Supplementary material for: Cerebral gray matter volume identifies healthy older drivers with a critical decline in driving safety performance using actual vehicles on a closed-circuit course
Source: Front Aging Neurosci. 2025 May 27;17:1462951. doi: 10.3389/fnagi.2025.1462951 (PMC12149418; doi:10.3389/fnagi.2025.1462951)
Supplement: Supplementary file 1 [file Data_Sheet_1.pdf]

Supplementary Data

Supplementary Figure

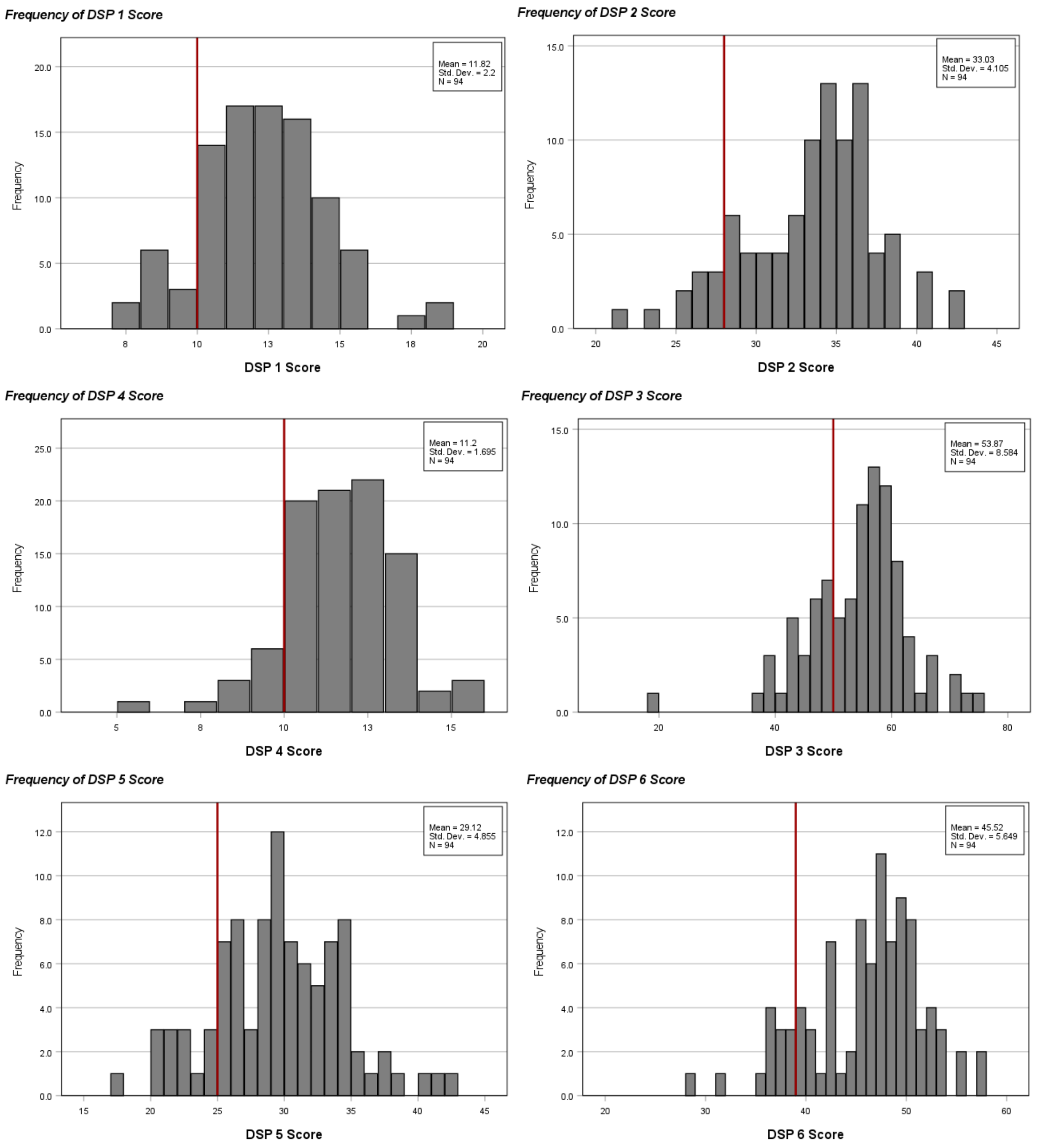

Supplementary Figure 1. Distribution of Driving Safety Performance (DSP) scores across six categories: DSP1 (visual search), DSP2 (speeding), DSP3 (signaling), DSP4 (vehicle stability), DSP5 (positioning), and DSP6 (steering). The red line on the x-axis indicates the 15th percentile threshold for each category, marking the boundary for the lowest 15% of scores.
